# Metabolomic and Lipidomic Profiling of Gliomas—A New Direction in Personalized Therapies

**DOI:** 10.3390/cancers14205041

**Published:** 2022-10-14

**Authors:** Magdalena Gaca-Tabaszewska, Joanna Bogusiewicz, Barbara Bojko

**Affiliations:** Department of Pharmacodynamics and Molecular Pharmacology, Faculty of Pharmacy, Collegium Medicum in Bydgoszcz, Nicolaus Copernicus University in Torun, 85-089 Bydgoszcz, Poland

**Keywords:** gliomas, metabolomics, lipidomics, pharmacotherapy, personalized medicine

## Abstract

**Simple Summary:**

Gliomas comprise an extremely diverse category of brain tumors that are difficult to diagnose and treat. As a result, scientists continue to search for new treatment solutions, with personalized medicine having emerged as a particularly promising therapeutic approach. Metabolomics and its sub-discipline, lipidomics, are two scientific fields well-suited to support this search. Metabolomics focuses on the physicochemical changes in the metabolome, which include all of the small endogenous and exogenous compounds in a biological system. As such, metabolic analysis can help identify important biochemical pathways which could be the targets for new therapeutic approaches. This review examines the new directions of personalized therapies for gliomas and how metabolomic and lipidomic analysis assists in developing these strategies and monitoring their effectiveness. The discussion of new strategies is preceded by a brief overview of the current “gold standard” treatment for gliomas and the obstacles that new treatment approaches must overcome.

**Abstract:**

In addition to being the most common primary brain tumor, gliomas are also among the most difficult to diagnose and treat. At present, the “gold standard” in glioma treatment entails the surgical resection of the largest possible portion of the tumor, followed by temozolomide therapy and radiation. However, this approach does not always yield the desired results. Additionally, the ability to cross the blood-brain barrier remains a major challenge for new potential drugs. Thus, researchers continue to search for targeted therapies that can be individualized based on the specific characteristics of each case. Metabolic and lipidomic research may represent two of the best ways to achieve this goal, as they enable detailed insights into the changes in the profile of small molecules in a biological system/specimen. This article reviews the new approaches to glioma therapy based on the analysis of alterations to biochemical pathways, and it provides an overview of the clinical results that may support personalized therapies in the future.

## 1. Introduction

Primary central nervous system (CNS) tumors account for 7% of deaths in people under 70, despite only making up 2% of all primary cancers [1]. In addition to being the most common type of primary brain tumor, gliomas are also among the most difficult to diagnose and treat [1]. At first, the classification of CNS tumors was based mainly on the tissue of origin, histological differences, and immunochemical tests; however, a 2016 recommendation from the World Health Organization (WHO) significantly extended the classification of gliomas by including the consideration of their molecular features, for instance, the presence or absence of an isocitrate dehydrogenase (IDH) mutation and 1p/19q codeletion [2]. In 2021, the classification of glioblastomas was further refined, leading to the identification of new subgroups. This new classification framework extended the role of molecular diagnostics while still considering histology and immunohistochemistry. Furthermore, the development of new molecular technologies enabled changes in both the nomenclature and grading of CNS tumors. Previous classifications used the tumor’s histological structure to determine its malignancy grade, which was traditionally expressed in Roman letters (grade I–IV). In contrast, the latest classification system expresses malignancy grades in Arabic numerals, and it is based on histological structure and additional factors like molecular alterations and natural history. One of the most aggressive and life-threatening grade 4 gliomas is glioblastoma (GBM). To date, around 30 types of these gliomas have been identified, with each subtype possessing a distinguishing genetic mutation [3]. Nonetheless, the treatment of gliomas remains a challenge, as the wide variety of gliomas, the tendency for late diagnosis, and the high diversity in the location of these tumors often make it difficult to achieve the desired results.

The current “gold standard” in glioma therapy is to remove the largest portion of the tumor that is safely possible via surgical resection, followed by temozolomide (TMZ) and radiation therapy. Temozolomide is an alkylating drug that is capable of crossing the blood-brain barrier and is usually well-tolerated by the patient. However, it is sometimes necessary to use reduced dosages in patients suffering from thrombocytopenia. Nevertheless, the resistance of cancer to TMZ still poses a challenge, making the standard therapy less than satisfactory. Moreover, researchers have postulated that increased patient survival rates could be obtained by resecting >/=98% of the tumor; unfortunately, this is highly difficult to achieve in many cases. Another FDA-approved drug for the treatment of recurrent gliomas is bevacizumab, which is an anti-angiogenic monoclonal antibody that works by attaching to vascular endothelial growth factor (VEGF), thereby inhibiting its ability to bind to receptors on the surface of endothelial cells. Researchers have theorized that the normalization of tumor vascularization via the reduction of interstitial pressure in the tumor will improve access to cytoreductive drugs. This has been supported by the findings of prior studies, which have shown that combining bevacizumab with conventional chemotherapy can increase both progression-free survival (PFS) and overall survival (OS) [4]. Subsequent studies examined the use of bevacizumab alongside the standard procedure with TMZ, finding that this approach prolonged PFS but not OS [5]. Hence, researchers continue to search for new glioma-treatment strategies and test existing therapies in clinical trials. 

One of the major problems for glioma-treatment drugs is the blood-brain barrier (BBB), which is a structure formed by endothelial cells that is fundamental to the protection of brain tissue. However, the BBB also restricts the delivery of drugs to the brain, which means that many oncologic drugs like alkylating agents (e.g., lomustine, carmustine, and temozolomide) and monoclonal antibodies (e.g., bevacizumab) cannot cross this barrier at sufficient therapeutic concentrations. While this problem can theoretically be solved by administering higher doses of these drugs, the large number of inevitable side effects makes this solution impossible in practice [6]. Consequently, the search for new drug targets that consider the variety of biochemical pathways modified during the formation of glial tumors remains ongoing [7]. Thus, this review highlights the importance of metabolomic and lipidomic studies in identifying alterations in biochemical pathways and potential biomarkers and the application of metabolomic and lipidomic profiling to assess the efficiency of new therapeutic approaches.

## 2. Metabolic Changes in Gliomas

**Figure 1:**
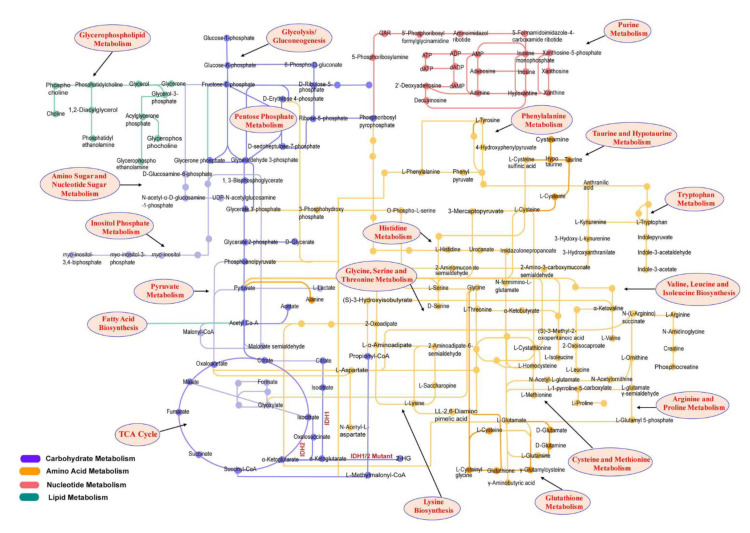
Major metabolomic pathways involved in brain tumor metabolism. The highlighted boxes represent the potential metabolic pathways involved in the brain tumor: glycolytic intermediates, glutamine, lipids, and TCA cycle metabolites significantly alter in malignant brain tumors. The accumulation of oncometabolite 2-HG as a result of the IDH1/2 mutant also contributes to malignancy. ADP, adenosine diphosphate; AMP, adenosine monophosphate; ATP, adenosine triphosphate; dADP, deoxyadenosine diphosphate, dAMP, deoxyadenosine monophosphate; dATP, deoxyadenosine triphosphate; CoA, coenzyme A; GAR; glycinamide ribonucleotide; IDH, isocitrate dehydrogenase; UDP, uridine diphosphate. Reprinted with permission from [7].

The diversity of gliomas is rooted in their origins, as well as in the area of the tumor from which the samples are taken. Usually, GBMs are characterized by a contrast-enhancing mass comprised of a highly cellular core surrounded by vascular proliferation. However, there is also a non-enhancing lesion around this core that may go undetected by magnetic resonance imaging (MRI); as such, samples from the non-enhancing margins of the tumor and tissue from the contrast-enhancing core can have completely different cellular and molecular compositions [8]. Gliomas are also characterized by remarkable biochemical plasticity when it comes to adapting to the microenvironment, which may lead to the development of treatment resistance [9]. Cancer cell metabolism is defined by many factors, such as increased glucose consumption, hypoxia, or immune cell infiltration, which should all be considered when determining the most appropriate treatment [7] (Figure 1).

When discussing metabolic reprogramming in glial tumors, carbohydrate metabolism alteration is the first process that should be considered. Thanks to the Warburg effect (described below), cancer cells undergo oxygen glycolysis instead of oxidative phosphorylation. This process converts pyruvate into lactate to produce NADPH, which can be used for the production of fuel biomass. Lactate, on the other hand, acidifies the microenvironment, facilitating tumor invasion. Moreover, glioma cells are constantly dividing and need to produce new nucleotides for new DNA replication and RNA transcription processes. Nucleotides are formed by glucose-6-phosphate, which is produced during glycolysis and introduced into the pentose-phosphate (PPP) pathway, where it is converted into ribose-5-phosphate. Furthermore, it is critical to consider the TCA (tricarboxylic acid) cycle, as it seems to be the center of energetic activity in cancer cells. The exemplary reverse TCA cycle in glioma cells is created by the conversion of citrate to pyruvate by ATP citric lyase and the subsequent conversion of pyruvate to oxaloacetate by pyruvate carboxylase. The resultant oxaloacetate can then be converted to p-enol pyruvate, which is a gluconeogenic precursor. Thus, alterations in the TCA cycle and the cellular mutations common to cancers have a unique impact on changes in the metabolism of amino acids (e.g., glutamine, valine, leucine, and isoleucine) and lipids, which will be discussed in greater detail below [10].

It is common knowledge that cancer cells metabolize glucose to produce energy. This phenomenon, known as the Warburg effect, is based on the non-oxidative glucose metabolism characteristic of cancer cells. However, findings have shown that glucose is not the only energetic substrate for glioma cells; rather, a large proportion of these cells’ energy also comes from fatty acid oxidation. Indeed, recent findings have shown that the metabolism of fatty acids via the Krebs cycle serves as the primary energy source for glioma cells [10]. Thus, the expression of two crucial components in fatty acid oxidation, namely, palmitoyltransferase 1 (CPT 1) and the SLC22A5, changes in gliomas. For this reason, agents such as etomoxir, which is a CPT 1 inhibitor, are considered in personalized glioma therapy [11,12]. Another possible target for glioma drugs may be sphingomyelin metabolism, as alterations in this process are a common feature of these tumors. It is well-known that, following radio- and chemotherapy, sphingomyelins are hydrolyzed by sphingomyelinases to ceramides, which induce apoptosis. However, cancer cells counter this by converting ceramides to sphingosine and then using sphingosine kinase (SK1 and SK2) to produce sphingosine-1-phosphate (S1P), which is responsible for cellular proliferation, angiogenesis, and motility. Another salvage pathway in the production of S1P is the conversion of sphingosine via acid ceramidase. Thus, all of these important enzymes appear to be good targets for treatments aimed at limiting cellular proliferation and migration. Notably, researchers have already developed drugs (e.g., carmofur) for such purposes that have been tested on gliomas but not in clinical trials or metabolomic research [13,14].

The findings of other trials have indicated that the topoisomerase II (TOP2) mechanism of action could be another good target for anti-glioma drugs. These ubiquitous enzymes are responsible for unlinking DNA strands and play a key role in many biological processes involving DNA, including anti-cancer activity [15]. For example, researchers have used in vitro cell models to test TOP2 drugs such as etoposide and doxorubicin, which initiate cellular apoptosis by damaging the cell’s DNA. Although crossing the BBB remains a problem, the anti-tumor activity of these drugs has been demonstrated in the literature [16].

*De novo* pyrimidine biosynthesis is another example of an alteration to a metabolic/genomic pathway during the formation of glioblastomas. A tumor’s growth is dependent on metabolic adaptations, which leads to increased *de novo* nucleotide biosynthesis. A number of specific enzymes (e.g., dihydroorotate dehydrogenase (DHODH) and uridine monophosphate synthetase (UMPS)) are known to be responsible for this process, as their activity allows ribosomal DNA transcription to be maintained in tumor cells. Recently, this knowledge has led to the targeting of DHODH to limit tumor growth and proliferation. Many trials have focused on DHODH inhibitors, such as the drug BAY 2402234, which, until now, has shown promising results for limiting glioma growth in cell line studies [17,18,19]. However, clinical trials have failed to produce significant results, and one trial begun in 2021 has been withdrawn (NCT05061251). As of today, the literature contains no metabolomics or lipidomics studies focusing on BAY 2402234.

Neurotransmitters can also be key components in both the neural and GBM microenvironments as they can serve pathological functions in gliomas, such as modulating tumor growth and progression. Thus, selected alterations in neurotransmitter levels during anti-glioma treatments will be examined in the following sections [20].

It is also worth noting that glioma cell apoptosis is dysregulated, which is a key reason for their prolonged survival. Thus, targeting specific components in the apoptotic pathway—for example, the p53 pathway, which plays a key role in regulating the mechanisms of the DNA damage response through apoptosis and cell cycle signaling, and IAPs, which are inhibitors of apoptosis proteins and the B-cell lymphoma family of proteins—can help in the search for new therapies [21]. Additionally, Lehtimaki et al. [22] observed metabolite changes in BT4C rat gliomas undergoing programmed cell death. Although their findings mainly showed increases in polyunsaturated fatty acids (PUFA), some alterations in metabolites such as glycine, taurine, and creatine were also observed [22].

In addition, key regulators of cellular bioenergetics such as 5′ Adenosine Monophosphate-Activated Protein Kinase (AMPK) and the phosphatidylinositol 3′-kinase (PI3K), protein kinase B (AKT), and the mammalian target of the rapamycin (mTOR) (PI3K/AKT/mTOR) signaling pathway should be mentioned as the attractive targets for novel anti-glioma drugs. Gliomas, most notably GBMs, alter energy metabolism to fuel proliferation, survival, and invasion. Thus, AMPK, as an mTOR and lipogenesis inhibitor, as well as the one ensuring viability through metabolic reprogramming in cancer cells, prolongs the life of cancer cells. When discussing gliomas, SBI-0206965, an aminopyrimidine derivative, should be considered as an AMPK inhibitory compound. According to Desai et al., SBI-0206965 has good brain permeability but low relative oral bioavailability, which may be due to its rapid hepatic metabolism. As this is a very recent study on rats, there are no clinical trials already ongoing [23]. Another cell study investigated apitolisib (GDC-0980)—a dual P13K/mTOR inhibitor, which has been already used in phase I/II studies in the treatment of solid tumors–which demonstrated the enhancement of glial cell apoptosis and the inhibition of tumor growth. Although there are no reports on utilizing metabolomics to study cellular bioenergetics regulator pathways, it is worth mentioning them because of their high importance in pharmacotherapy drug development. Moreover, as the above-mentioned studies are very recent, no clinical trials have been found [24].

### Metabolomics and Lipidomics

One of the first drugs developed to treat cancer was methotrexate, an antifolate drug that inhibits thymidine synthesis. Subsequently, scientists discovered that arresting cancer expansion by targeting alterations in the biochemical pathways was a good approach to drug development [25]. However, targeted therapies for gliomas still lag far behind those of other forms of cancer. This disparity is partially related to the lack of screening biomarkers specific to gliomas, which causes these tumors to be diagnosed relatively late compared to other types of cancer, such as prostate, hepatocellular, or colorectal cancer, for which PSA, AFP, and CEA respectively provide good specificity and sensitivity [26].

Many trials have been conducted with the goal of developing targeted therapies for specific alterations in the biochemical pathways [9]. However, this approach limits the scope of the searched metabolites. As such, an increasing amount of attention is being devoted to metabolomics research, as this approach enables the discovery of new diagnostic biomarkers and, consequently—after the analysis of the altered biochemical pathways—new grip points for personalized therapies. Metabolomics refers to the targeted and untargeted profiling of the whole metabolome, including both endogenous and exogenous metabolites [7]. Recently, lipid analysis was distinguished from metabolomics as a new and distinct area of science known as lipidomics [27]. There are many methods that can be used for metabolomic and lipidomic analysis. Currently, mass spectrometry (MS) is the dominant analytical platform used for metabolomics and lipidomics due to its extremely specific level of detection at pico- and nanomolar concentrations. Although mass spectrometry is usually coupled with separation-based methods like liquid (LC) or gas (GC) chromatography, the literature also contains reports of the use of direct infusion MS, with electrospray ionization (ESI) being the most common format [28]. Data obtained via metabolomics provide comprehensive information about physicochemical changes at the metabolome level, which may take place during the development of various diseases, as well as the metabolome’s response to stimuli (i.e., environmental factors, diet, and lifestyle changes). Such global insight can help to define important pathways that could be targeted for new therapeutic options [7]. 

Metabolomics also overlaps with other -omics sciences like genomics, transcriptomics, and proteomics. Although these approaches all interact with one another, metabolomics is the best suited for studying alterations in the cellular phenotype due to its specific focus on biochemical changes [29]. Perhaps the best example of the correlation between genomics and metabolomics is genetic alteration/mutation, which can be observed in the metabolic pathways. With respect to gliomas, a mutation in the portion of the genome encoding the IDH enzyme results in the formation of oncometabolite D-2-hydgoxyglutarate (D-2-HG) instead of α-ketoglutarate. This mutation is now used to grade gliomas, as it mainly occurs in tumors with lower malignancy grades. However, the grade of a glioma does not only rely on the presence/absence of IDH mutation; rather, it also relies on the results of histological and immunochemistry tests. Given the complexity of the coexisting features, attempts have been made to characterize glioma phenotypes that reflect all of the alterations present at different biochemical levels. It has previously been reported that changes in the metabolic pathways of IDH mutant (mIDH) gliomas involve both lipids and polar metabolites. Fack et al. [30] showed how mIDH gliomas affect the levels of phospholipids, including phosphatidylethanolamine (PE), phosphatidylinositol (PI), phosphatidylserine (PS), phosphatidylcholine (PC), and phosphoglycerol (PG) (Table 1). Subsequently, Miyata et al. [31] observed a decrease in metabolites in the tricarboxylic acid (TCA) cycle and diminished carnitine levels related to the β-oxidation pathway in IDH mutants [30,31]. In another example, increased levels of the oncometabolite, D-2-hydgoxyglutarate, were observed in cells harboring the IDH mutation, which consequently led to clinical trials exploring the efficacy of drugs targeting the mIDH cells [32,33]. 

At present, there are many metabolites that can be used to distinguish glioma tissue from healthy controls or determine malignancy grades. For example, choline is a potential biomarker that can be used to distinguish neoplastic tissue from healthy tissue, while amino acids like alanine, valine, proline, and glutamate have been found to be useful for discriminating glioma grades [34]. Goryńska et al. [35] reported several metabolites, including amino acids, nucleotides, and acylcarnitines, that could be used to discriminate the IDH wildtype from mutants, 1p19q codeletion from the wildtype, and various malignancy grades of gliomas [35]. In a recent review, Pienkowski et al. [36] discuss metabolites that have emerged as significant for the characterization and diagnosis of gliomas [36]. 

As previously discussed, metabolomics enables endogenous metabolite profiling and is a good phenotypic tool for obtaining information about the current pathophysiological status of patients. Moreover, metabolomics allows us to observe all of the individual alterations in a patient relating to genetics, environmental influences, or medical history, as well as pharmacokinetic and pharmacodynamic drug processes, which, when considered together, can enable the selection of appropriate drugs and dosages. The branch of metabolomics responsible for this latter analysis is known as pharmacometabolomics. Pharmacometabolomics is an extremely important metabolomic approach in relation to personalized medicine, as it enables scientists to predict a patient’s drug response based on the interaction between the drug pharmacology and the patient’s pathophysiology. Metabolomics is a diagnostic methodology that enables deeper insights into changes in tumor biochemistry, which can be critical in determining the most appropriate therapy; in contrast, pharmacometabolomics is better conceived of as a prognostic methodology. The goal of personalized medicine is to use patient-specific data to develop customized therapeutic approaches that maximize the effectiveness of the treatment while minimizing side effects [37,38]. 

## 3. Challenges in Glioma Therapy—Blood-Brain Barrier

Although some drugs are effective against gliomas when tested *in vitro*, they typically obstruct the blood-brain barrier (BBB) when monitored *in vivo*. The BBB is an extremely important structure that separates the brain tissue from the circulatory system in order to regulate the transport of molecules. There are many BBB-specific transporters responsible for delivering hydrophilic compounds to the brain cells and discharging neurotransmitters and their precursors from the brain to the blood [51]. Due to the close connections between the capillary endothelial cells and the efflux activity of ABC (ATP-binding cassette) transporters, such as P-glycoprotein, the BBB prevents the diffusion of 100% of macromolecular drugs and 98% of small-molecule drugs [6,30]. Thus, since efficient drug delivery by the BBB is critical to the development of glioblastoma therapy, researchers are currently conducting trials to assess various invasive and noninvasive methods of crossing through the BBB. Although invasive methods such as brain microdialysis, intracerebral implantation, and intraventricular delivery are effective at penetrating the BBB, they can damage the surrounding healthy tissue and cause adverse effects. On the other hand, noninvasive methods such as prodrugs, BBB permeability modulation, nanotechnologies, and receptor-mediated transport are more appealing options, as they do not carry such risks [52] (Table 2). One of the most promising approaches for delivering therapeutic agents across the blood-brain barrier is nanotechnology with the use of liposomes, which are compatible with both hydrophilic and lipophilic molecules [53]. The results of Ananda et al. [54] phase II clinical trial of temozolomide and pegylated liposomal doxorubicin in the post-operation treatment of patients with glioblastoma were satisfying with respect to safety, but no significant clinical benefits were observed regarding 6-month progression-free survival (6PFS) and overall survival (OS) [54]. Moreover, a more recent study involving the multifunctional targeting of epirubicin and resveratrol in mice models showed improved transport across the BBB and higher survival rates among animals with brain tumors [55].

## 4. Gold Standard Therapy

The “gold standard” in glioma treatment is surgery followed by temozolomide (TMZ) chemotherapy and radiation. TMZ is the most popular chemotherapeutic, especially in first-line treatment, as it is a lipophilic, alkylating drug that can effectively cross the blood-brain barrier. However, only about 20% of the administered TMZ ultimately reaches the brain tissue and attempts to use increased doses have resulted in the amplification of its toxic effects [56]. TMZ’s mechanism of action is based on the creation of cytotoxic forms of guanine and adenine, O^6^-methylguanine (O^6^-MG), N^7^-methylguanine (N^7^-MG), and N^3^-methyladenine (N^3^-MA), which, in turn, cause cell apoptosis. TMZ is administered as a prodrug and undergoes intracellular conversion into the potent methylating agent, MTIC, after BBB penetration. MTIC methylates the nucleotides, mostly guanine, and creates their cytotoxic forms [57]. It has been reported that treatment with TMZ is not effective for all glioma patients because growing resistance to the drug has led to increased treatment failure. Thus, the mechanism of resistance is strongly connected with O^6^-methylguanine-DNA methyl-transferase (MGMT), as this enzyme is responsible for repairing DNA and, consequently, methylated/cytotoxic lesions. Hence, only patients with methylated MGMT have a better chance of long-term survival after TMZ treatment. Moreover, the MGMT promoter hypermethylation is currently the only biomarker for TMZ response in patients with gliomas [58,59]. Although TMZ is not the best choice for all glioma patients, it is still one of the very few options. As such, new clinical trials aimed at improving treatment with TMZ (instead of replacing it) are currently ongoing (NCT03932981, NCT00424554). 

**Figure 2:**
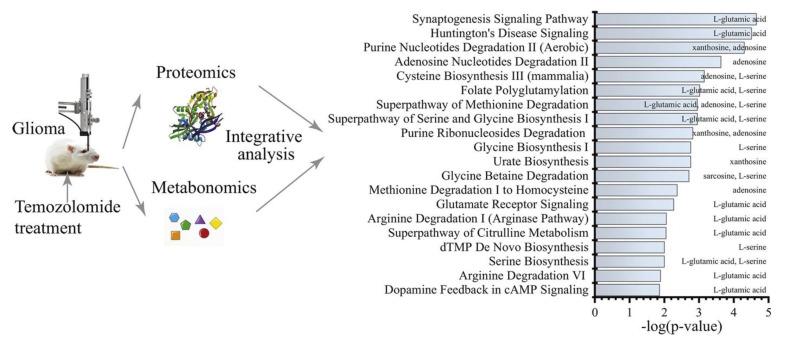
Changes in metabolome and proteome under the influence of temozolomide. Reprinted from [40] with permission.

Even though TMZ’s mechanism of action is well-known, metabolomics studies have been conducted to identify the biomarkers of the effectiveness of TZM therapy. In one study, St-Coeur et al. [43] conducted an in vitro comparison of TMZ-sensitive and TMZ-resistant cell lines treated with TMZ with an MGMT-inhibitor (lomeguatrib) and TMZ alone; the findings showed that the dysregulation of the concentration of the metabolites was dependent on the selected treatment and cells. To confirm the observed differences in the metabolomes of the TMZ-sensitive and TMZ-resistant cell lines, St-Coeur et al. profiled the tumor samples obtained from the patients. A detailed description of these studies is presented in Table 1 [44]. Menglin et al. [40] also successfully applied metabolomic profiling in a rat glioma model treated with TMZ. In this work, the authors identified approximately 251 proteins and nine endogenous metabolite markers in the brain that significantly changed after TMZ treatment. As a result, some of these metabolites were proposed as potential biomarkers [40] (Figure 2). 

The literature contains many studies that employ metabolomic and lipidomic profiling to compare TMZ-sensitive and TMZ-resistant cells. These studies are described in detail in Table 1.

Researchers have also examined TMZ resistance based on drug repurposing studies. Drug repurposing is a strategy wherein drugs already approved by the FDA—thus, avoiding first-phase clinical trials—are safely marketed for a different purpose. According to Valtorta et al. [42], metformin (MET)—a drug from the biguanides group commonly used to treat type II diabetes—has good synergic activity with TMZ. The research on TMZ-sensitive cell lines (U251) showed that treatment combining TMZ with metformin has a positive influence on glioma cell resistance to TMZ and overcomes TMZ resistance in T98G-TMZ-resistant cell lines. Moreover, Valtorta et al. found that combining TMZ and MET significantly slowed glioma cell growth and produced metabolomic alterations, such as changes in the levels of nucleotides, amino acids, lipids, and glutathione [42]. 

Another example of a drug that has been repurposed for the treatment of TMZ-resistant gliomas is olaparib, which is a poly ADP ribose polymerase (PARP) inhibitor usually used to treat patients with BRCA1 or BRCA2 mutations, such as breast cancer. According to Zampieri et al. [60], TMZ resistance is not primarily related to the MGMT enzyme activity; rather, it is the effect of the presence of fitter mitochondria with higher oxidative phosphorylation activity. Consequently, olaparib, which is also an inhibitor of mitochondrial complex I, has been proposed as a combined therapy for inhibiting DNA repair and impairing cancer cell respiration, thus also inhibiting TMZ resistance. Unfortunately, the literature does not contain any metabolomic studies examining the use of olaparib to treat glioblastomas [60].

The metabolomics studies examining the use of temozolomide conducted by Valtorta et al. [42] and others are presented in Table 1. 

## 5. Immune Therapy—Monoclonal Antibodies

**Figure 3:**
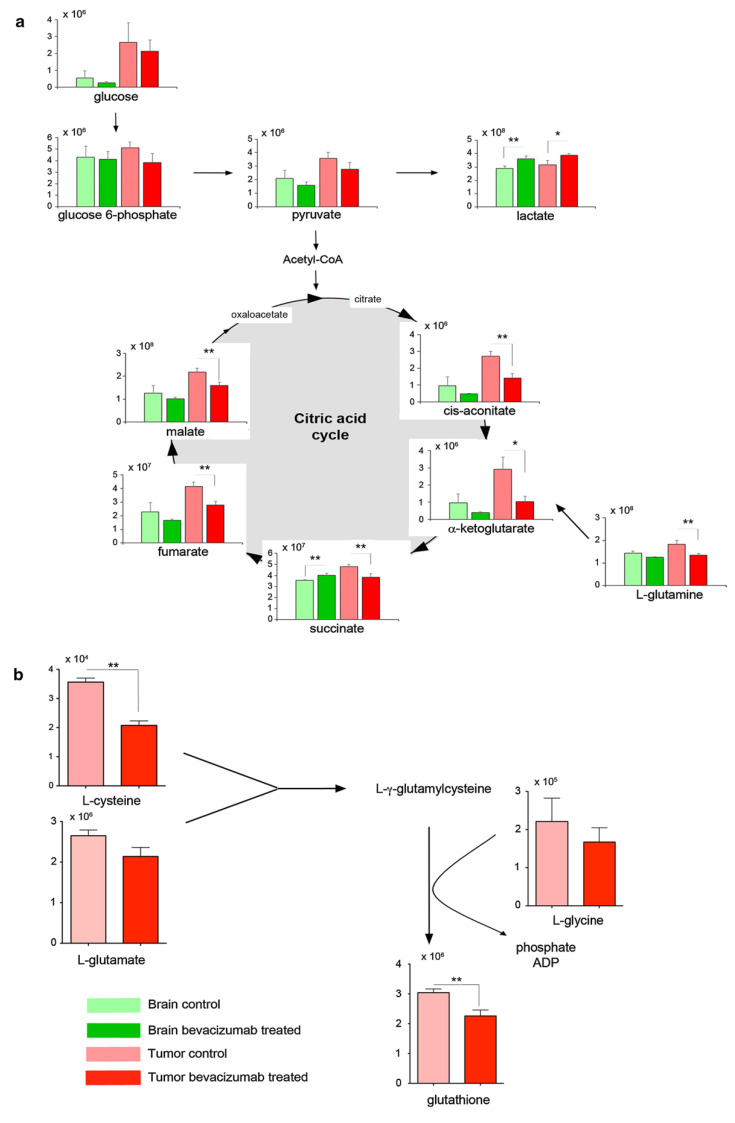
Reduction in metabolites associated with the TCA cycle after bevacizumab treatment. (**a**) Total metabolite levels (unlabeled and labeled) were quantified by LC–MS analysis. In addition to decreased glucose, glucose-6-phosphate and pryruvate levels, a reduction of metabolites associated with the TCA cycle was measured in the bevacizumab-treated tumors. These included pyruvate, cis-aconitate, α-ketoglutarate, succinate, fumarate and malate. Moreover, reduced levels of l-glutamine were observed following bevacizumab treatment. (**b**) Bevacizumab treatment led to reduced levels of glutathione and metabolites associated with glutathione synthesis, including l-cysteine, l-glutamate and l-glycine. Reprinted from [48] under the terms of the Creative Commons.

When considering monoclonal antibodies, bevacizumab, a humanized anti-VEGF antibody, is one of the WHO-approved drugs for treating various neoplasms. Bevacizumab has demonstrated very good efficiency in the treatment of various cancers, including renal cancer, non-small-cell lung cancer, metastatic breast cancer, and especially metastatic colorectal cancer, where it has been shown to significantly increase patient survival rates when added to standard chemotherapy [61]. Due to their high degree of malignancy, gliomas proliferate very rapidly and, hence, build up very quickly. Bevacizumab inhibits angiogenesis, which improves the progression-free survival of glioblastoma patients; however, the OS of these patients remains very short [5,62]. In monitoring the metabolic fingerprint of bevacizumab’s effects, Fack et al. [48] observed a shift from aerobic to anaerobic metabolism in glioma cells (Figure 3). For instance, induced hypoxia in the tumor cells and increased glycolysis with the activation of the pentose phosphate pathway (PPP) were observed. Moreover, bevacizumab treatment led to a reduction in metabolites associated with the tricarboxylic acid cycle (TCA); a reduction in total glucose, glucose-6-phosphate, pyruvate, cis-aconitate, α-ketoglutarate, succinate, fumarate, and malate resulted in increased lactate production. In addition, Fack et al. [48] also reported the down-regulation of the γ-glutamyl cycle and a reduction in all associated metabolites, including cysteine, glutamate, glycine, and glutathione [48]. Most of the above-mentioned findings were confirmed in mutant IDH1 glioma cells by Mesti et al. [47], who found significant changes not only in the metabolites from the glutamine group (e.g., glutamate, alanine, and glycine) but also in the lipids. In this work, Mesti et al. observed modified levels of polyunsaturated fatty acids (PUFA), glycerophosphorylcholine (GPC), and phosphatidylcholine (PC) following bevacizumab treatment and considered these changes early markers of metabolic alterations due to this treatment [47]. The identification of biochemical pathways affected by bevacizumab enables the proposition of potentially effective combination therapies. 

A detailed description of all metabolomics studies examining the use of bevacizumab is presented in Table 1. 

In 2017, Wick et al. [63] conducted a phase-2 randomized trial focusing on the use of lomustine, an alkylating drug, in conjunction with bevacizumab to treat patients with recurrent glioblastoma. The main goal of this study was to assess the overall survival of the participants, but a secondary goal was to examine the methylation status of the MGMT promoter. The results showed no significant differences between treatment with lomustine alone and treatment with lomustine and bevacizumab [63]. Another phase I trial demonstrated the safety of an approach combining bevacizumab and irinotecan for the treatment of recurrent glioblastoma. Moreover, the findings of this study also revealed improvements in the 6-month progression-free survival of the participants. Despite these results, findings have yet to demonstrate that bevacizumab possesses direct antitumor properties or the ability to prolong OS in glioma patients [64]. Nonetheless, other monoclonal antibodies have recently been tested as potential anti-glioma drugs. For instance, studies investigating pembrolizumab and nivolumab, two anti-PD1 immune checkpoint inhibitors, have yielded findings indicating their therapeutic effectiveness in solid tumors with mismatch repair deficiency (MMRd). In fact, pembrolizumab has been approved by the US Food and Drug Administration (FDA) for the treatment of this kind of tumor. Elsewhere, a pilot study by Lombardi et al. aimed to establish the role of MMRd status as a potential biomarker of pembrolizumab efficiency. However, pembrolizumab did not exhibit good activity in recurrent high-grade gliomas (HGGs) in patients with complete or partial loss of MMRd expression. As a result, the authors suggested that the combination of pembrolizumab and various drugs that target CD68+ macrophages could be a good area for further research [65]. At present, researchers are conducting trials examining the effectiveness of pembrolizumab in combination with other drugs, such as cyclophosphamide (another alkylating drug) and bevacizumab (NCT05366062).

## 6. IDH Inhibitors and Epigenetic Therapies for Treatment IDH-Mutant Glioma 

As previously discussed, IDH mutation is a critical element of glioma classification. Indeed, according to the WHO, 70% of grade 2 and 3 gliomas are characterized by this mutation [66]. Metabolomic and lipidomic studies play a key role in the detection of potential biomarkers, such as 2-hydroxyglutarate (2-HG), which is a substrate arising in glioblastomas with the IDH mutation. Thus, new, non-invasive methods of detecting levels of 2-HG prior to treatment have been developed to extend the efficiency therapeutic approaches [67]. Magnetic resonance spectroscopy (MRS) is an example of one such approach that has been proposed for this purpose, as it possesses low risk, good repeatability, and the ability to probe many tumor regions at one time, thus allowing clinicians to select the best therapy path [68].

This rich body of research on IDH mutants has led to the development of FDA-approved IDH mutant inhibitors (mIDH1 inhibitor), which have been used for the treatment of other diseases, such as acute myeloid leukemia (AML). Using MRS, researchers have demonstrated that the effectiveness of these drugs is largely based on their ability to reduce the oncometabolite, 2-HG. With findings that confirm that FDA-approved drugs, like ivosidenib (mIDH1 inhibitor) and enasidenib (mIDH2 inhibitor), induce clinical and molecular remission in AML, further clinical trials on these two and other isocitrate dehydrogenase inhibitors for gliomas were initiated [69]. For instance, Mellinghoff et al. [70] phase I clinical trials on ivosidenib revealed that this drug is well tolerated, especially in treating non-contrast-enhancing gliomas and was able to provide prolonged disease control while also reducing growth rates [70]. In another study, Mellinghoff et al. indicated that vorasidenib, an IDH 1/2 mutant inhibitor, possessed a favorable safety profile for the treatment of non-enhancing gliomas. Their findings showed that vorasidenib had a median PFS of 31 months and was generally well-tolerated by the patients [71]. In contrast, clinical trials for the mIDH inhibitor, enosidenib, are still ongoing, with the results obtained thus far being unsatisfactory (NCT02273739). 

At present, the literature lacks research investigating the effectiveness of these drugs via metabolomic and lipidomic fingerprinting; however, based on previous studies of mIDH and wIDH (IDH-wild-type) tumors, it is suggested that 2-HG would be a perfect biomarker candidate. For this reason, the literature tends to contain research aimed at monitoring specific oncometabolites rather than metabolomic studies. 

Moreover, there are also drugs that counteract the epigenetic changes common to IDH-mutant gliomas, such as histone deacetylase inhibitors, bromodomain inhibitors, poly-ADP ribose polymerase inhibitors, and nicotinamide phosphoribosyl transferase inhibitors. However, the results obtained from research focusing on this group are still indeterminate, with some studies needing to be terminated due to excessively high risks of side effects and drug toxicity [66].

## 7. Carnitine Palmitoyl Transferase-1a (CPT-1a)-Inhibitor—Etomoxir 

**Figure 4:**
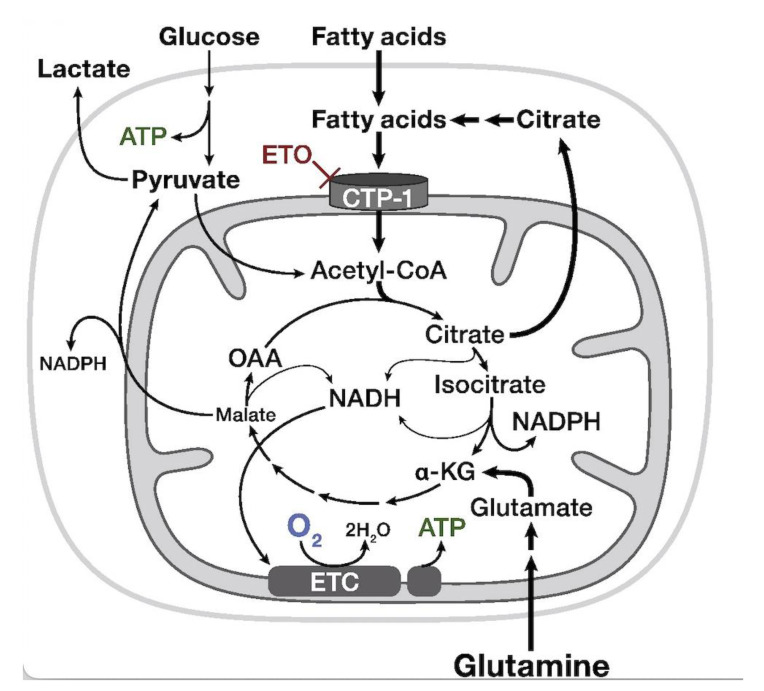
Illustration of fatty acid oxidation and synthesis in mitochondria. Fatty acids enter the mitochondria for β-oxidation to Acetyl CoA. Acetyl CoA enters the TCA cycle generating NADH. The NADH is then oxidized by the ETC, consuming oxygen or producing NADPH, as indicated. Endogenous fatty acids can be synthesized de novo from glucose or glutamine. Glucose is converted to pyruvate via a series of catabolic reactions, and the resultant pyruvate is either converted to lactate or enters the mitochondria to be converted to Acetyl CoA. Acetyl CoA enters the TCA cycle and exits as citrate, which is then exported to cytosol for the synthesis of fatty acids. Glutamine enters the TCA cycle after conversion to α-ketoglutarate (α-KG) and exits as malate and citrate into the cytosol for fatty acid synthesis. ETO: etomoxir, a CPT-1a inhibitor. Reprinted with permission from [75].

As previously mentioned, the growth and proliferation of cancer cells requires high amounts of energy. This energy can be sourced either from glucose metabolism or fatty acid oxidation (FAO), which both lead to the formation of new ATP molecules. FAO is strongly integrated with the carnitine shuttle system, which plays a major role in transporting activated long-chain fatty acids to the β-oxidation sites in the mitochondria. In addition, FAO also helps to stabilize cell membranes by removing long-chain acyl-CoA and excess acyl groups from the body [72]. One of the enzymes responsible for transporting fatty acids across the mitochondrial membrane is carnitine palmitoyl transferase-1 a (CPT-1a). Many studies, including those focused on gliomas, have proposed FAO as a very good potential target for new drugs. As such, researchers have considered etomoxir as a CPT-1a inhibitor, as it slows the proliferation of glioma cells and prolongs the patient’s time of survival [73]. The metabolomic data relating to the use of etomoxir to inhibit CPT-1a shows a reduction in NADPH levels and the induction of oxidative stress in the targeted glioma cells. In addition, CPT-1 inhibitors block the process responsible for converting carnitine and acyl-CoA into acylcarnitine and CoA (Figure 4). This leads to alterations in the levels of fatty acids and acylcarnitines, which, in turn, decelerates tumor proliferation. Moreover, acylcarnitine can be proposed as a potential biomarker for etomoxir treatment [50,74]. Additionally, researchers have previously reported carnitine and acylcarnitine as discriminant metabolites for differentiating IDH-wildtype and IDH-mutant gliomas. Given these findings, metabolomic research related to FAO metabolism and the carnitine shuttle system has recently expanded in the hopes of identifying new treatment strategies. 

A detailed description of all metabolomic studies focusing on etomoxir is provided in Table 1. 

Promising results have been obtained from clinical trials examining the combined use of etomoxir and reradiation to target hypoxia and improve treatment efficiency for cancers such as lung adenocarcinoma or prostate cancer [76]. In addition, Taib et al. [77] observed the significant inhibition of cell proliferation when etomoxir was applied alongside oleic acid in GBM and astrocytic cells [77]. To date, most studies in this area have employed in vitro experiments using gliomas or other cancer cells; however, the literature also contains clinical trials and animal studies demonstrating the inhibitory effects of etomoxir treatment in neurodegenerative diseases, such as Parkinson’s or Alzheimer’s disease [78]. Thus, we can assume that BBB crossing is an issue that can be overcome in the case of etomoxir and that more trials examining the use of this drug to treat gliomas are forthcoming.

## 8. Topoisomerase-II Inhibitors

**Figure 5:**
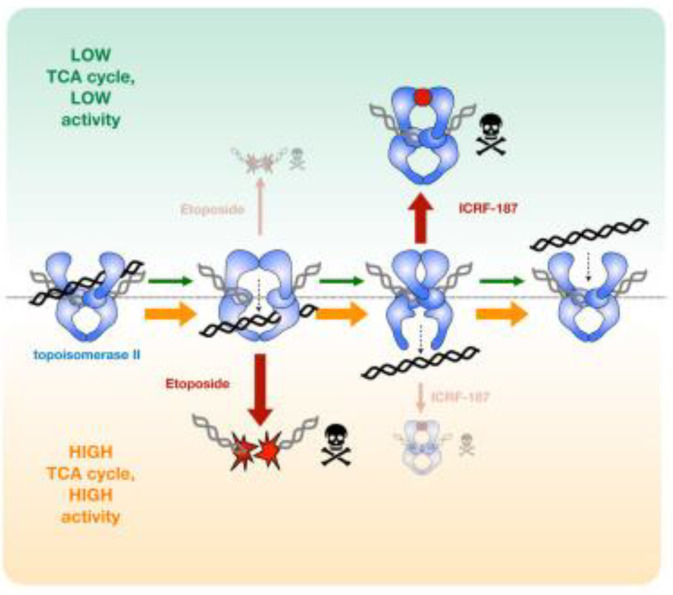
The metabolomic connection between the TCA cycle and TOP-2 inhibitor activity. When TCA cycle flux is low (green) topo II strand passage activity is not stimulated. General catalytic inhibitors (e.g., ICRF-187) that decrease topo II activity are more effective in this state, as compared with a high TCA state (orange). By contrast, topo II poisons (e.g., etoposide) are more toxic in the high TCA state because elevated topo II activity leads to increased formation of DNA cleavage complexes. Reprinted with permission from [79].

DNA topoisomerases (TOPs), mostly IIA, are proven targets of anticancer drugs, which typically act through topoisomerase poisoning, thereby arresting the formation of replication forks and double-strand fractures. This approach is controversial, as high doses may lead to cardiotoxicity or even to the development of new cancers [15]. To improve methods based on the use of TOP-2 inhibitors so as to enable their targeted use, it is extremely important to examine changes in the metabolome. For instance, Lee et al. [79] discovered a strong connection between the TCA cycle and the mechanism of action of TOP-2; specifically, they found that the mechanism of action of TOP-2 is controlled by the activity of TCA cycle metabolites. The stimulating effect of TCA metabolites on TOP-2 can be observed, for example, in citrate and isocitrate, which chelate the Mg^2+^ ions required for the catalytic activity of TOP-2. Moreover, it has also been noted that changes in the TCA cycle flux in cells impacts TOP-2 activity and drug response. The connection between cellular metabolism and DNA control can significantly impact targeted therapy with TOP-2 inhibitors [79] (Figure 5). Etoposide is a notable topoisomerase IIA poison that has shown very promising results in in vitro and preclinical studies on GBM cells and was subsequently tested in clinical trials for patients with recurrent glioma. However, in their phase II clinical trials, Reardon et al. found that the combined use of etoposide and bevacizumab did not result in any significant improvement in patients’ conditions compared to the use of bevacizumab alone. Instead, they observed that this combination treatment resulted in a high level of toxicity [80]. In 2021, Chen et al. [81] applied etoposide and carboplatin (alkylating drug) to treat patients with recurrent glioma, as this combination of drugs is widely used in the treatment of malignant tumors. Unfortunately, their results showed that this combination therapy did not offer any advantages compared to the use of high doses of temozolomide, as demonstrated in the RESCUE Study [82]. However, the studies of Chen et al. resulted in one significant observation: the disease control rate (DCR)—which is the percentage of patients with advanced or metastatic cancer who have achieved complete response, partial response, and stable disease to a therapeutic intervention in clinical trials of anticancer agents—time to progression (TTP), and overall survival (OS) were significantly longer in patients with grade 2 and 3 gliomas compared to patients with grade 4 gliomas [81].

As mentioned above, high doses of TOP-2 poisons are toxic to human organisms, while doses that are too small may not affect gliomas, especially higher-grade gliomas, which creates difficulty with regards to crossing the BBB. For that reason, numerous attempts have been made to improve the BBB permeability of drugs targeting different diseases of the brain. For instance, Wei et al. [83] assessed focused ultrasound (FUS) as a new approach for enhancing drug delivery by opening the BBB. FUS has been an FDA-approved method for the unilateral treatment of essential tremors since 2016 and for tremor-dominant Parkinson’s disease since 2018. In 2021, the FDA further approved FUS for the treatment of patients with advanced Parkinson’s disease, with clinical trials focusing on its use to treat chronic pain currently ongoing. [84].

FUS is predicated on directing sound waves through the skull to a specific site indicated by MRI or CT, thereby avoiding the need for a craniectomy. In the most recent study, the concentration of etoposide in brain tissue was assessed after the use of FUS. The results of this in vitro experiment were very promising, as this combination therapy reduced tumor growth by as much as 45% and prolonged the median overall survival rate. However, the experiment must be performed in vivo to confirm the effectiveness of the approach, as direct extrapolation of the results from the in vitro studies is not reliable [83,85]. Convection-enhanced delivery (CED) is another method of enhancing BBB crossing that has been tested with etoposide. CED is a local drug-delivery technique that uses hydraulic pressure to bypass the BBB to allow the direct delivery of the drug to the target area, thus enabling better distribution [86]. In one study, Sonabend et al. applied CED to the intratumoral delivery of a high dose of etoposide, with results showing that this approach increased the drug’s anti-tumor effect on the proneuronal subtype of GBM [87]. In summary, the use of TOP-2 poisons/inhibitors is very promising and a forward-thinking method, but it still requires some improvements to achieve its desired outcomes. 

## 9. Other Approaches

Dichloroacetate (DCA) is a pyruvate dehydrogenase kinase (PDK) inhibitor that was originally used to treat children with congenital mitochondrial disorders. Thus, PDK can inhibit puryvate dehydrogenase, which can, in turn, lead to the targeting of glucose metabolism. Consequently, DCA has become a topic of considerable interest in cancer treatment. According to Cook et al. [88], dichloroacetate leads to the radiosensitization of high-grade gliomas, which improves the effectiveness of radiotherapy. Thus, by inhibiting puryvate dehydrogenase, DCA reverses the Warburg effect and activates mitochondrial oxidative phosphorylation at the expense of glycolysis [88]. 

With regards to metabolomic influence, the ketogenic diet has been proposed recently as a glioma therapy or a method of enhancing treatment. The ketogenic diet is based on significantly reducing the body’s supply of sugar, which forces it to obtain its energy mainly from fats and proteins. Thus, researchers theorize that this diet may lead to a reduction in glycolysis. Stanford et al. conducted the first in vitro studies on this topic in 2010, with numerous additional clinical trials either currently ongoing or having been documented in the literature [10,89]. The ongoing clinical trials examining the effectiveness of the ketogenic diet in treating gliomas include: (NCT04461938), (NCT04691960), and (NCT04730869).

## 10. Conclusions

Despite dynamic and wide-ranging research into glioma, patient outcomes remain poor in terms of their overall survival, particularly with respect to relapses and life quality. Therefore, this review specifically focused on metabolomic and lipidomic studies, as these approaches allow researchers to monitor the changes in the small molecules in the tumor and the organism as a whole, which can reveal potential new targets for drugs and enable the assessment of the effects of new therapies on both tumors and healthy tissues. 

## Figures and Tables

**Table 1:** Summary of the influence of different glioma therapies on changes in the metabolome and lipidome.

Drug	Goal	Studied Species	Biological Material	Analytical Platform	Significant Metabolites	Altered Metabolic Pathways	Reference
Temozolomide	The effect of the complex of formononetin and calycosin on glioma temozolomide sensitivity	Rat, injected with C6 cells	C6 cell lines	GC-MS	Treatment is related with - ↓aspartic acid, - ↓glutamic acid, - ↓serine, - ↓methionine, - ↓ribitol, - ↓phosphonic acid, - ↓ribose. In the group with combined treatment compared to the group treated only with temozolomide.	- Aminoacyl-tRNA biosynthesis, - Arginine biosynthesis, - Alanine, aspartate, and glutamate metabolism, - Cysteine and methionine metabolism, - D-glutamine and D-glutamate metabolism. * searched by authors, *p* < 0.05 and impact > 0.05	[39]
Temozolomide	The effect of the complex of formononetin and calycosin on glioma temozolomide sensitivity	Rat, injected with C6 cells	Serum	GC-MS	Treatment is related with - ↓ornithine, - ↓oxalacetic acid, - ↓alanine, - ↓glycine, - ↓phenylalanine, - ↓serine, - ↓proline, - ↓succinic acid, - ↓creatinine, - ↓mannose, - ↓propionic acid, - ↓glutaric acid, - ↓lactic acid. In the group with combined treatment compared to the group treated only with temozolomide.	- Aminoacyl-tRNA biosynthesis, - Alanine, aspartate, and glutamate metabolism, - Glyoxylate and dicarboxylate metabolism, - Citrate cycle, - Glutathione metabolism, - Glycine, serine, and threonine metabolism, - arginine and proline metabolism. * searched by authors, *p* < 0.05 and impact > 0.05	[39]
Temozolomide	The effect of the complex of formononetin and calycosin on glioma temozolomide sensitivity	Rat, injected with C6 cells	Glioma tissue	GC-MS	Treatment is related with: - ↓xanthosine, - ↓glutamic acid, - ↓ornithine. In the group with combined treatment compared to the group treated only with temozolomide.	- Arginine biosynthesis. * searched by authors, *p* < 0.05 and impact > 0.05	[39]
Temozolomide	Response to temozolomide treatment	Rat, injected with C6 cells	Plasma	LC-MS	Treatment is related with - ↑adenine, - ↑cis-9,10-epoxystearic acid, - ↑citraconic acid, - ↑glucose, - ↓acetylneuraminic acid, - ↓sarcosine. In TMZ-treated group compared to the control group (without treatment).	- Glycine, serine, and threonine metabolism, - D-Glutamine and D-glutamate metabolism, - Purine metabolism, - Alanine, aspartate, and glutamate metabolism. ** searched by authors, *p* < 0.05 and impact > 0.4	[40]
Temozolomide	Response to temozolomide treatment	Rat, injected with C6 cells	Tumor tissue	LC-MS	Treatment is related with - ↑adenosine, - ↑acetylornithine, - ↑sarcosine, - ↑serine, - ↑xanthosine, - ↑acetyl-serine. In TMZ treated group compared to the control group (without treatment).	- Purine metabolism, - Glycine, serine, and threonine metabolism, - D-Glutamine and D-glutamate metabolism, - Alanine, aspartate, and glutamate metabolism. ** searched by authors, *p* < 0.05 and impact > 0.4	[40]
Temozolomide	Response to temozolomide treatment	Mouse, injected with GL261 cells	Tumor tissue	NMR	Treatment using TMZ is related to changes in: - mobile lipids, - polyunsaturated fatty acids in mobile lipids, - N-acetylaspartate, - creatine. In TMZ-treated group compared to the control group (without treatment).	…	[41]
Temozolomide	The effect of metformin on glioma temozolomide sensitivity	Mouse, injected with TMZ-sensitive U251 and TMZ-resistant T98G cells	Tumor tissue	GC-MS	The application of TMZ with metformin compared to treatment with TMZ alone: - TMZ-sensitive cells: significant reduction of 32 metabolites. - TMZ-resistant cells: significant changes in 8 compounds: nucleotides, amino acids, glutathione, lipids, amino sugars, and β-alanine metabolism.	- Amino acids, fatty acids, and lipid metabolism, - Amino acids pathway.	[42]
Temozolomide	Response to temozolomide treatment	---	Cell lines (TMZ-sensitive and TMZ-resistant U373 cells)	NMR	Treatment using TMZ is related with: - ↑arginine, - ↑leucine, - ↑myo-inositol, - ↑taurine - ↑valine, In TMZ-sensitive group compared to the TMZ-resistant group. Combination of TMZ with lomeguatrib is related with: - ↓citrate and - ↓succinate, - ↓citric acid cycle intermediates, - ↓glutathione. In TMZ-sensitive group compared to the TMZ-resistant group. Independently to used treatment: - ↓alanine, - ↓choline, - ↓creatine - ↓phosphorylcholine. In TMZ-sensitive group compared to the TMZ-resistant group.	Treatment using TMZ is related with: - Aminoacyl-tRNA biosynthesis, - Valine, leucine, and isoleucine biosynthesis, - Valine, leucine, and isoleucine degradation, - Ascorbate and aldarate metabolism, - Taurine and hypotaurine metabolism, - Arginine biosynthesis. Combination of TMZ with lomeguatrib: - Citrate cycle (TCA cycle), - Alanine, aspartate, and glutamate metabolism, - Butanoate metabolism, - Propanoate metabolism. Independently to used treatment: - Glycine, serine, and threonine metabolism, - Glycerophospholipid metabolism. *** manually searched, *p* < 0.05	[43]
Temozolomide	TMZ-sensitive and TMZ-resistant glioblastoma profiling	Human	Brain tumor tissue	NMR	Treatment using TMZ is related with: - ↓adenosine, - ↑alanine, - ↓glucose, - ↓glutathione, - ↑isoleucine. In the TMZ sensitive group compared to the TMZ-resistant group.	- Aminoacyl-tRNA biosynthesis, - Valine, leucine, and isoleucine biosynthesis. *** manually searched, *p* < 0.05	[43]
Temozolomide	Response to temozolomide treatment	---	Cell lines (U87)	LCMS	Treatment using TMZ is related with: - ↑acyl-carnitine, - ↑trans-2-Enoyl-CoA, - ↑3-hydroxyacyl-CoA, - ↑acetyl-CoA, - ↑citrate, - ↑cis-aconitate, - ↑α-ketoglutarate, - ↑succinate, - ↑malate, - ↓Pamitoyl-L-carnitine - ↓Acetyl-Lcarnitine, - ↓citric acid, - ↓cis-aconitic acid, - ↓fumaric acid, - ↓L-malic acid. In the TMZ-sensitive group compared to the TMZ-resistant group.	- Citrate cycle (TCA cycle), - Alanine, aspartate, and glutamate metabolism, - Glyoxylate and dicarboxylate metabolism, - Butanoate metabolism, - Pyruvate metabolism, - Fatty acid elongation, - Arginine biosynthesis, - Propanoate metabolism, - Synthesis and degradation of ketone bodies, - D-Glutamine and D-glutamate metabolism. *** manually searched, *p* < 0.05	[44]
Temozolomide	Profiling the metabolome of TMZ-sensitive and TMZ-resistant cells	---	Cell lines (NSP—TMZ resistant; U87M)	LC-MS	Response to TMZ doses: - ↑intracellular succinate to α-ketoglutarate ratio along with ↑ TMZ dose, - ↓succinate, - ↑α-ketoglutarate, - ↓ornithine, - ↓pyruvate. In the TMZ-sensitive group compared to the TMZ-resistant group. TMZ effect on intracellular metabolome: - ↓glutamine, serine, tryptophan, - ↑succinate, malate, citrate/isocitrate and ascorbate. In the TMZ-sensitive group compared with the TMZ-resistant group. TMZ effect on extracellular metabolome: - ↑succinate, ornithine, histidine and uric acid succinate, malate, citrate/isocitrate and ascorbate, - ↓arginine, asparagine and glutamate tryptophan. In the TMZ-sensitive group compared with the TMZ-resistant group.	Response to TMZ doses: - Alanine, aspartate, and glutamate metabolism, - Arginine biosynthesis, - Citrate cycle (TCA cycle), - Aminoacyl-tRNA biosynthesis - Glyoxylate and dicarboxylate metabolism, - D-Glutamine and D-glutamate metabolism, - Arginine and proline metabolism. TMZ effect on intracellular metabolome: - Butanoate metabolism, - Nitrogen metabolism, - Histidine metabolism, - Pyruvate metabolism, - Glutathione metabolism. TMZ effect on extracellular metabolome: - Citrate cycle (TCA cycle), - Aminoacyl-tRNA biosynthesis, - Alanine, aspartate, and glutamate metabolism, - Glyoxylate and dicarboxylate metabolism, - Arginine biosynthesis, - Arginine and proline metabolism, - Butanoate metabolism. *** manually searched, *p* < 0.05	[45]
Temozolomide	Profiling the metabolome of TMZ-sensitive and TMZ-resistant cells	---	Cell lines (TMZ sensitive and resistant 8MBGA) medium	LC-MS, GC-MS	Treatment using TMZ is related with: - ↑LysoPC(16:0), - ↑LysoPE(16:0), - ↑Indoleacetaldehyde, - ↑palmitic acid, - ↑stearic acid, - ↑aminomalonate, - ↑citric acid, - ↓glutamic acid, In the TMZ-sensitive group compared with the TMZ-resistant group.	- Lipid metabolism, - Tryptophan metabolism, - Heme synthesis pathway.	[46]
Bevacizumab	Assessment of bevacizumab effectiveness	---	Cell lines (mIDH1-U87)	NMR	Treatment is related with: - ↑2OH-glutarate, - ↑glycerylphosphorylcholine/choline, - ↑glutamate, - ↑alanine, - ↑ (CH2)n-CH3, - ↑ (CH 2)n-CH2-(CH2)m, - ↑creatine, - ↑creatine, - ↑choline, - ↑Taurine, - ↑glycine, - ↑glycerylphosphorylcholin. In the treated group compared with a control group (without treatment).	- Glycine, serine and threonine metabolism, - Aminoacyl-tRNA biosynthesis, - Glutathione metabolism, - Alanine, aspartate, and glutamate metabolism, - Porphyrin and chlorophyll metabolism, - Glyoxylate and dicarboxylate metabolism, - Glycerophospholipid metabolism, - Arginine and proline metabolism, - Primary bile acid biosynthesis, - Nitrogen metabolism, - D-Glutamine and D-glutamate metabolism, - Taurine and hypotaurine metabolism. *** manually searched, *p* < 0.05	[47]
Bevacizumab	Response to bevacizumab treatment	Human	Tumor tissue	LC-MS	Treatment is related with: - ↓cysteine, - ↓glutamate, - ↓glycine, - ↓glutathione. In treated group compared with a control group (without treatment).	- Glutathione metabolism, - Aminoacyl-tRNA biosynthesis, - Porphyrin and chlorophyll metabolism, - Glyoxylate and dicarboxylate metabolism, - Glycine, serine, and threonine metabolism, - Nitrogen metabolism, - D-Glutamine and D-glutamate metabolism, - Thiamine metabolism, - Taurine and hypotaurine metabolism, - Arginine biosynthesis, - Butanoate metabolism, - Histidine metabolism, - Pantothenate and CoA biosynthesis. *** manually searched, *p* < 0.05	[48]
Etomoxir	Assessment of etomoxir effectiveness	---	Cell lines (BT549)	LC-MS	Treatment is related with: - changes in acylcarnitine species.	- Fatty acid oxidation.	[49]
Etomoxir	Assessment of etomoxir effectiveness	---	Cell lines (Mesenchymal (MES83, MES326, and MES1027A) and proneural (PN19, PN84))	LCMS	Treatment is related with: - ↓medium and long-chain acylcarnitines. In the treated group compared with a control group (without treatment).	- Fatty acid oxidation.	[50]

- ↑ up-regulation of metabolites levels. ↓ down-regulation of metabolites levels.

**Table 2:** Invasive and noninvasive methods of crossing the blood-brain barrier.

Invasive Methods	Noninvasive Methods
Brain microdialysis	Prodrugs
Intracerebral implantation	BBB permeability modulation
Intraventricular delivery	Nanotechnologies
	Receptor mediated transport

## Data Availability

Not applicable.
